# Health of Students

**Published:** 1860-11

**Authors:** 


### HEALTH OF STUDENTS.

AMHERST COLLEGE, through the agency of gentlemen who have been admirers and readers of the JOURNAL OF HEALTH for some years, has established a Professorship of "Hygiene and Physical Education," and have appointed to the chair, Dr. John W. Hooker, the worthy son of Worthington Hooker, M.D., of New-Haven. Prof. Hooker brings to his aid the learning and the skill acquired from the enjoyment of the highest advantages in Europe and America for perfecting his medical studies and researches ; hence we may confidently look for success in this first official and practical endeavor to make the health of students a matter of systematic attention, an indispensable branch of study in a collegiate course, and as necessary to a diploma as a proficiency in the languages or mathematics. It is now thirty-two years since, in a series of letters, we urged the establishment of a similar chair in a flourishing college, but "the time was not yet." One of the first objects in commencing the publication of this JOURNAL was to place the knowledge of the means of preserving the health within easy reach of all students, especially of theological students, as will be seen by reading the prospectus in the first number. If therefore any reader has a son ready for college, send him at once to Amherst College, in the Old Bay State, if, while you have an ambition that your child shall become a scholar, you have the wisdom and the humanity to arrange that he shall graduate in robust health and live a life of enjoyment and usefulness instead of passing his weary years in tantalizing inefficiency and wretched invalidism.

